# Synergy of diffraction and spectroscopic techniques to unveil the crystal structure of antimonic acid

**DOI:** 10.1038/s41598-021-97147-0

**Published:** 2021-09-07

**Authors:** S. F. Mayer, J. E. Rodrigues, I. Sobrados, J. Gainza, M. T. Fernández-Díaz, C. Marini, M. C. Asensio, J. A. Alonso

**Affiliations:** 1 Instituto de Ciencia de Materiales de Madrid (ICMM), Consejo Superior de Investigaciones Científicas (CSIC), Sor Juana Inés de la Cruz 3, 28049 Madrid, Spain; 2 European Synchrotron Radiation Facility, ESRF, 71 Avenue des Martyrs, 38043 Grenoble, France; 3 Institut Laue Langevin (ILL), BP 156X, 38042 Grenoble, France; 4 CELLS-ALBA Synchrotron, Cerdanyola del Valles, 08290 Barcelona, Spain

**Keywords:** Materials science, Materials for energy and catalysis

## Abstract

The elusive crystal structure of the so-called ‘antimonic acid’ has been investigated by means of robust and state-of-the-art techniques. The synergic results of solid-state magic-angle spinning nuclear magnetic resonance spectroscopy and a combined Rietveld refinement from synchrotron X-ray and neutron powder diffraction data reveal that this compound contains two types of protons, in a pyrochlore-type structure of stoichiometric formula (H_3_O)_1.20(7)_H_0.77(9)_Sb_2_O_6_. Some protons belong to heavily delocalized H_3_O^+^ subunits, while some H^+^ are directly bonded to the oxygen atoms of the covalent framework of the pyrochlore structure, with O–H distances close to 1 Å. A proton diffusion mechanism is proposed relying on percolation pathways determined by bond-valence energy landscape analysis. X-ray absorption spectroscopy results corroborate the structural data around Sb^5+^ ions at short-range order. Thermogravimetric analysis and differential scanning calorimetry endorsed the conclusions on the water content within antimonic acid. Additional 0.7 water molecules per formula were assessed as moisture water by thermal analysis.

## Introduction

One of the long-standing chemical identity issues is that corresponding to the so-called ‘antimonic acid’ (hereafter: AA), also referred to as hydrated antimony pentoxide (frequently termed HAP), antimony oxide hydrates, and Sb(V) hydroxide. This material has proven useful for a variety of applications due to its distinctive proton-conducting nature^1–4^, ionic exchangeability^5–10^, and radiation resistance^7^, serving as potential candidate for photocatalyst^11,12^, fuel cells electrolyte^13,14^, heavy metals remover^15,16^, and for its application in electrochromic displays^3^ or as precursor of several useful derivatives^7,11,17–19^, among others. This enigmatic substance was already described by J.J. Berzelius. In his pioneering work in 1812, Berzelius published^20^ the preparation of a ‘hydrated antimony pentoxide’ by treating alkali antimonate with diluted nitric acid, while his junior student H. Rose synthesized it in 1824 by hydrolysis of antimony pentachloride^21^. One of the earliest structural discussions concerning AA salts dates back from the mid-nineteenth century, where oxygen/metal ratios were elucidated, and six-sided crystals of magnesium, cobalt, and nickel antimonates are successfully synthesized^22^. More than a 100 years later, the atomic arrangement within the crystal of this elusive compound (or more properly speaking, series of compounds) is still being a topic of debate. Perhaps the first modern review gathering all these results is the one presented by J. W. Mellor^17^ in 1929. The most accepted formula for AA is Sb_2_O_5_·*x*H_2_O; depending on the synthesis and water content; water amounts varied from one to six H_2_O molecules per formula unit. A few years later, L. Pauling suggested the formula HSb(OH)_6_, equivalent to that of Sb_2_O_5_·7H_2_O, as the most likely composition for the acid phase^23^. This was supported by the water amount in the crystals and the ionic radius and behaviour of the Sb^5+^ cation, and it was widely accepted as a base of further compositional and structural determinations^7,24–27^. So forth, although researchers took many efforts to define the composition of AA, the crystal structure was totally unknown, perhaps given the difficulty of obtaining well-crystallized samples.

In a big attempt to shed light on the plausible structures adopted by AA-type compounds, Abe and Ito^28^ synthesized AA samples from antimony pentachloride under different acidic conditions, temperatures, and aging times, obtaining solids tagged as amorphous, glassy, and crystalline solids^1^. They demonstrated that the crystalline phase can be fostered at higher acid concentrations, longer aging times, and suitable aging temperatures, regardless of the starting phase employed. The crystalline solid, of formula unit Sb_2_O_5_·4H_2_O, was defined in the $Fd\overline{3 }m$ space group, with a lattice constant of 10.38 Å. This was the first time that a pyrochlore-type phase was effectively defined for the AA crystalline oxides. With 6 days of difference, Baetsle and Huys announced similar results for AA samples obtained from K and Na antimonates, with similar unit-cell parameters and same compositions^5^, also concomitant with pioneering properties predicted for hydrated Sb_2_O_5_^29^.

In the present work, we have chosen a straightforward synthesis procedure, to our best knowledge described for the first time by Ozawa et al*.*^3^, yielding well-crystallized powder samples from easy-to-handle reactants, namely Sb_2_O_3_ and H_2_O_2_. It has been characterized by state-of-the-art techniques including neutron and synchrotron X-ray diffraction (NPD and SXRD, respectively), solid-state magic-angle spinning nuclear magnetic resonance spectroscopy (MAS NMR) and local order techniques like X-ray absorption spectroscopy (XAS), in complement with thermogravimetric analysis and differential scanning calorimetry (TGA/DSC), as well as scanning electron microscope (SEM). Our findings agree on a consistent picture of the atom-scale arrangement of AA, in a pyrochlore-type framework containing both H_3_O^+^ and H^+^ units accounting for the acidic behaviour of this material.

## Results and discussion

### Long-range order structural determinations

**Figure 1:**
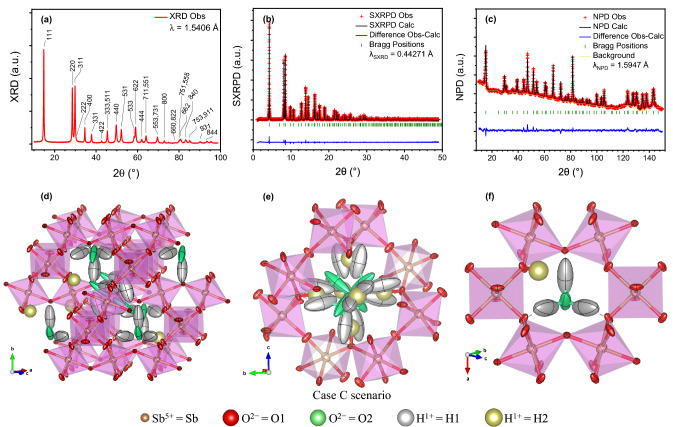
Diffraction patterns and structural representation of AA. (**a**) Laboratory XRD diagram of (H_3_O)_1.20(7)_H_0.77(9)_Sb_2_O_6_ (λ = 1.5406 Å Cu Kα radiation) with peaks indexed in a face-centred cubic unit cell with *a* = 10.36052(15) Å. (**b**,**c**) Plots from the combined Rietveld refinement from SXRD and NPD data. Experimental (red crosses), calculated (solid black line), and difference (solid blue line at the bottom) (**b**) SXRD (λ_SXRD_ = 0.44271 Å) and (**c**) NPD (λ_NPD_ = 1.5947 Å) patterns, with Bragg reflection positions marked by vertical green bars. (**d**–**f**) Views of the AA pyrochlore final structure (‘Case C’ scenario described in the Supporting Information). Atoms of the three panels are presented as anisotropic displacement ellipsoids at a 95% probability level. Caption colour reference at the bottom of Figure is common to all figures. (**d**) Representation of a snapshot of the crystal approximately along the $[\overline{1 }01]$ direction: the covalent framework made by the (Sb^5+^O_6_) corner-sharing octahedra consists of 16*d* (Sb, brown) and 48*f* (O1, red) fully occupied sites, with golden (H2) protons linked to the skeleton O1 atoms at distances of 1.13(6) Å. Hydronium subunits are constituted by silver (H1) protons bonded to green (O2) oxygens at 1.323(11) Å in a tetrahedral distribution (with 104.0(16)° angles). These atoms present high mobility, with prolate anisotropic ellipsoids oriented along the (*x*,*x*,*x*) main threefold diagonal. (**e**) Close up of the statistical distribution of a single cavity wherein the O2 and H1 are distributed at 32*e* and 96*g* Wyckoff positions, respectively, with SOFs close to 0.301(2), and H2 atoms at 48*f* sites exhibiting a SOF of 0.129(15). Only 1.20(7) H_3_O^+^ groups and 0.77(9) H2 species are statistically present in each cavity. (**f**) Snapshot of a single octahedra-sharing crown with a hydronium subunit and a H2 proton. In dotted lines: H1 atoms of the H_3_O^+^ subunit establish H bonds with the O1 atoms at about 1.400(10) Å.

Crystalline AA powder was obtained in the form of a colloidal slurry by soft-chemistry procedures as described in “Methods”. The obtained compound of nominal formula (H_3_O)_*p*_H_2−*p*_Sb_2_O_6_ and defect pyrochlore-type crystal structure has been investigated using long- and short-range characterization techniques, in order to determine its composition and atomic arrangement. Firstly, a peak indexing over a laboratory XRD pattern was performed as shown in Fig. 1a, confirming that AA adopts a cubic pyrochlore-type structure belonging to the $Fd\overline{3 }m$ (# 227, ${O}_{h}^{7}$) space group, in agreement with previous reports^3,28^. Briefly, pyrochlore-type oxides present a general formula *A*_2_*B*_2_O_6_O′, where *A* is a voluminous mono-, di- or trivalent cation, *B* is a smaller metal of higher oxidation state, and O′ is an oxygen atom that can be partially or even entirely absent, giving rise to the so-called defect pyrochlores *A*_2_*B*_2_O_6_O′_1−δ_. When O′ is present, two structural groups can be identified within the unit cell: a main covalent framework of *B*_2_O_6_ corner-sharing octahedra, and a sub-lattice *A*_2_O′, interspersed in a tetrahedral arrangement. Once the XRD data of AA were indexed in the $Fd\overline{3 }m$ space group, the crystalline structure was fully determined by a combined Rietveld refinement from SXRD and NPD data collected at room temperature. Patterns from NPD and SXRD data contained sharp diffraction peaks, consistent with a cubic pyrochlore phase with *a* = 10.36052(15) Å, as displayed in Fig. 1b,c. An origin choice # 2 at $\overline{3 }m$ was adopted for this analysis. The in-detail refinement procedure is described in the Supplementary Information, while the refinement parameters can be found in “Methods”.

The final refinement includes Sb and O1 atoms located at 16*d* ($${\raise0.5ex\hbox{$\scriptstyle 1$} \kern-0.1em/\kern-0.15em \lower0.25ex\hbox{$\scriptstyle 2$}}$$,$${\raise0.5ex\hbox{$\scriptstyle 1$} \kern-0.1em/\kern-0.15em \lower0.25ex\hbox{$\scriptstyle 2$}}$$,$${\raise0.5ex\hbox{$\scriptstyle 1$} \kern-0.1em/\kern-0.15em \lower0.25ex\hbox{$\scriptstyle 2$}}$$) and 48*f *(*x*,$${\raise0.5ex\hbox{$\scriptstyle 1$} \kern-0.1em/\kern-0.15em \lower0.25ex\hbox{$\scriptstyle 8$}}$$,$${\raise0.5ex\hbox{$\scriptstyle 1$} \kern-0.1em/\kern-0.15em \lower0.25ex\hbox{$\scriptstyle 8$}}$$) Wyckoff sites, constituting the covalent Sb_2_O_6_^2−^ framework of the pyrochlore oxide. Two types of H atoms were identified: H1 located at 96*g* (*x*,*x*,*z*) sites, constituting together with the O2 atoms at 32*e* (*x*,*x*,*x*) the hydronium H_3_O^+^ units, and H2 species located at 48*f *(*x*,$${\raise0.5ex\hbox{$\scriptstyle 1$} \kern-0.1em/\kern-0.15em \lower0.25ex\hbox{$\scriptstyle 8$}}$$,$${\raise0.5ex\hbox{$\scriptstyle 1$} \kern-0.1em/\kern-0.15em \lower0.25ex\hbox{$\scriptstyle 8$}}$$) positions, directly linked to the Sb_2_O_6_ covalent framework. O2 can be considered to be the O′ atoms of the standard pyrochlore structure. Ultimately, the individual atomic anisotropic displacement factors for each non-equivalent atom were determined, with the only exception of the H2 species, which was modelled as an isotropic sphere. This is due to a strong divergence of the model when attempted to be anisotropically determined, probably due to its vicinity to the resonant H1 atom. Attempts to incorporate additional water or hydronium molecules within the structure, as for example centred at 8*b* sites occupying the empty cages generated by the main framework or along the main diagonal (as reported by Slade et al*.*^30^), resulted in atomic site occupation factors (SOFs) close to zero, many times even reaching negative isotropic displacement factors. This way, tens of feasible structural alternatives and atomic incorporations were discarded, many of which containing constrains or restrains varying O–H bond length distances and O2/H1,2 SOF ratios. No preferred orientation is expected to occur due to the isotropy of the cubic structure and the octahedral shape of AA microcrystals, and neither the parameters of preferred orientation nor roughness were refined as they showed no improvement in the reliability factors.

Occupancies of H1, H2, and O2 atoms were constrained to each other according to the MAS NMR analysis, and strongly endorsed by a TGA/DSC study, as explained in the water and proton content assessment section; occupancy constrains partially relied on the electroneutrality of the crystal structure. For the Sb and O1 elements, small displacement ellipsoids were obtained (*U*_eq_ = 0.98(2) × 10^−2^ and 0.83(10) × 10^−2^ Å^2^, respectively), denoting small vibrational and delocalization of the scattering contributions. In contrast, although far smaller than those obtained in the ‘Case B’ scenario described in the Supplementary Information, O2 and H1 atoms belonging to the hydronium units exhibited rather large cigar-shaped (prolate type) displacement ellipsoids of *U*_eq_ = 5.4(4) × 10^−2^ and 6.7(8) × 10^−2^ Å^2^, stretched along the (*x*,*x*,*x*) direction. They are reminiscent to those described in the (H_3_O)_1+*p*_Sb_1+*p*_Te_1−*p*_O_6_ series^31^. This is expected, considering their high multiplicity and their relatively small occupancy, as well as by the fact that the hydronium units may rattle within the wide cavities generated by the Sb_2_O_6_^2−^ framework, where a high diffusivity of the hydronium units across its channels is expected. Finally, H2 atoms presented somewhat in-between isotropic displacements (*U*_iso_ = 3.626(1) × 10^−2^ Å^2^), suggesting lower ionic mobility and lower atomic delocalization. The final structure achieved by Rietveld refinement of both SXRD and NPD diffraction data for the AA is exhibited in detail in Fig. 1d–f. Besides, an animated representation of the H_3_O^+^ units and H2 protons in their Sb_2_O_6_^2−^ framework cavity is displayed in Supplementary File 2, and its caption in Supplementary File 1. A powder diffraction CIF file is also available as Supplementary Information.

At room temperature, convergence *R*_*B*_ Bragg R-factors achieved values of 3.55% for SXRD and 2.47% for NPD, suggesting that the calculated model fits reasonably well with the experimental data. To summarize, all these agreement factors, together with the final atomic positions, anisotropic and isotropic equivalent displacement factors, and the SOF of each species are recapitulated in Table 1.

**Table 1:** Structural parameters for AA refined from combined SXRD and NPD data. Unit-cell (*a*), fractional atomic coordinates (*x*, *z*), Debye–Waller anisotropic (*u*_*ij*_) and equivalent isotropic (*U*_eq_) displacement factors, SOFs and Rietveld agreement factors (R_p_, R_wp_, R_exp_, χ^2^, and R_Bragg_) for (H_3_O)_1.20(7)_H_0.77(9)_Sb_2_O_6_, with cubic space group $Fd\overline{3 }m$ (*#* 227) and *Z* = 8, from dual SXRD and NPD data refinement collected at 298 K (λ_SXRD_ = 0.44271 Å, λ_NPD_ = 1.5947 Å, Origin Choice # 2).

Pyrochlore	Antimonic acid	
*a* (Å)	10.36073(9)	
*V* (Å^3^)	1112.17(2)	
**Sb, 16*d* (**$${\raise0.5ex\hbox{$\scriptstyle {\mathbf{1}}$} \kern-0.1em/\kern-0.15em \lower0.25ex\hbox{$\scriptstyle {\mathbf{2}}$}}$$,$${\raise0.5ex\hbox{$\scriptstyle {\mathbf{1}}$} \kern-0.1em/\kern-0.15em \lower0.25ex\hbox{$\scriptstyle {\mathbf{2}}$}}$$,$${\raise0.5ex\hbox{$\scriptstyle {\mathbf{1}}$} \kern-0.1em/\kern-0.15em \lower0.25ex\hbox{$\scriptstyle {\mathbf{2}}$}}$$**)**	**Sb**^**5+**^	
*u*_11_ = *u*_22_ = *u*_33_^a^	0.01034(15)	
*u*_12_ = *u*_13_ = *u*_23_^a^	0.0008(2)	
*U*_eq_ (Å^2^)	0.01034(15)	
SOF	1.0000	
**O1, 48*f* (*x*,**$${\raise0.5ex\hbox{$\scriptstyle {\mathbf{1}}$} \kern-0.1em/\kern-0.15em \lower0.25ex\hbox{$\scriptstyle {\mathbf{8}}$}}$$**,**$${\raise0.5ex\hbox{$\scriptstyle {\mathbf{1}}$} \kern-0.1em/\kern-0.15em \lower0.25ex\hbox{$\scriptstyle {\mathbf{8}}$}}$$**)**	**O**^**2−**^	
*x*	0.43095(17)	
*u*_11_^a^	0.0038(13)	
*u*_22_ = *u*_33_^a^	0.0113(9)	
*u*_23_^a,b^	− 0.0059(9)	
*U*_eq_ (Å^2^)	0.0088(10)	
SOF	1.0000	
**O2, 32*e* (*x,x,x*)**	**O**^**2−**^	
*x*	0.0652(7)	
*u*_11_ = *u*_22_ = *u*_33_^a^	0.055(4)	
*u*_12_ = *u*_13_ = *u*_23_^a^	0.043(4)	
*U*_eq_ (Å^2^)	0.055(4)	
SOF	0.301(17)	
**H1, 96*g* (*x*,*x*,*z*)**	**H**^**+**^	
*x*	0.3321(8)	
*z*	0.0602(17)	
*u*_11_ = *u*_22_^a^	0.033(4)	
*u*_33_^a^	0.139(16)	
*u*_12_^a^	0.011(6)	
*u*_13_ = *u*_23_^a^	− 0.022(8)	
*U*_eq_ (Å^2^)	0.068(8)	
SOF	0.301(2)	
**H2, 48*f* (*x*,**$${\raise0.5ex\hbox{$\scriptstyle {\mathbf{1}}$} \kern-0.1em/\kern-0.15em \lower0.25ex\hbox{$\scriptstyle {\mathbf{8}}$}}$$**,**$${\raise0.5ex\hbox{$\scriptstyle {\mathbf{1}}$} \kern-0.1em/\kern-0.15em \lower0.25ex\hbox{$\scriptstyle {\mathbf{8}}$}}$$**)**	**H**^**+**^	
*x*	0.323(4)	
*U*_eq_ (Å^2^)	0.036261(10)	
SOF	0.129(15)	

Reliability factors	SXRD	NPD
R_p_ (%)	6.85	0.893
R_wp_ (%)	10.5	1.22
R_exp_ (%)	5.53	1.86
χ^2^	3.60	0.433
R_Bragg_ (%)	3.55	2.47

^a^Anisotropic *u*_ij_ (×10^4^).

^b^***u***_**12**_ = ***u***_**13**_ = 0.

**Figure 2:**
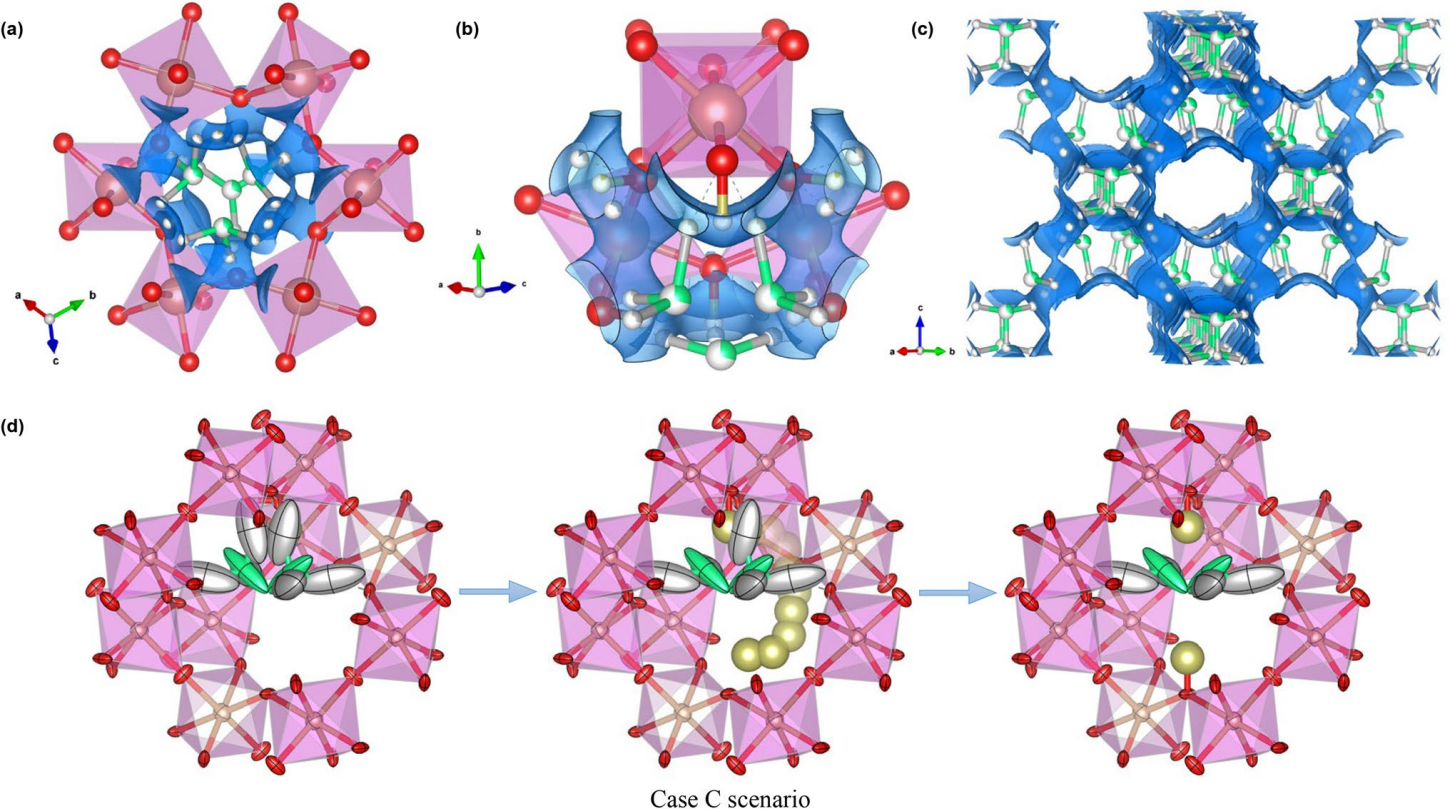
Proton percolation pathway and mechanism in the AA (‘Case C’ scenario described in the Supplementary Information). Atoms in all six panels are presented as anisotropic displacement ellipsoids at a 95% probability level. The colour reference is the same one used in Fig. 1. (**a**,**b**) Close up of the AA final structure; the ionic percolation path is highlighted in blue. The pathway involves both H^+^ species in a 3D interconnected arrangement, generating the 3D percolation isosurface defined for the AA. The ionic percolation begins with a low percolation activation energy barrier of 0.13 eV. (**c**) Proton percolation pathway in a simplified view of the AA unit cell approximately along the $$[110]$$ direction. For the sake of clarity, the Sb_2_O_6_^2−^ octahedra-sharing covalent framework is hidden, and only the H_3_O^+^ acid groups and the H2 species are visible. Statistically, less than a third of the hydronium units here figured are present. (**d**) Proposed ionic migration mechanism of two H1 atoms from 96*g* to 48*f* (H2) Wyckoff sites, for those cages wherein two H_3_O^+^ subunits coexist. The repulsive electrostatic forces between the two O2 species would force their shift away from the 8*a* ($${\raise0.5ex\hbox{$\scriptstyle 1$} \kern-0.1em/\kern-0.15em \lower0.25ex\hbox{$\scriptstyle 8$}}$$,$${\raise0.5ex\hbox{$\scriptstyle 1$} \kern-0.1em/\kern-0.15em \lower0.25ex\hbox{$\scriptstyle 8$}}$$,$${\raise0.5ex\hbox{$\scriptstyle 1$} \kern-0.1em/\kern-0.15em \lower0.25ex\hbox{$\scriptstyle 8$}}$$) along the (*x*,*x*,*x*) direction. One of the in-between protons is shared between the two O2 atoms and the O1 framework oxygen, while the other is relocated at the antipodal position of the cavity by following the percolation path highlighted in panels (**a**–**c**), achieving an electrostatic stability.

The crystallographic formula obtained from the structural refinement is (H_3_O)_1.20(7)_H_0.77(9)_Sb_2_O_6_, which presents electroneutrality within the standard deviation and is also endorsed by the low Rietveld reliability factors and the reasonable equivalent isotropic displacement factors obtained for each mobile atom. It is interesting to examine the ionic conduction path obtained from a bond-valence energy landscape (BVEL) study of the crystal, illustrated in Fig. 2a–c. The conduction pathway, highlighted with a blue isosurface, involves both types of H atoms and exhibits an isotropic distribution along the three axes. The percolation of the H atoms presents a very low activation energy of 0.13 eV and a noticeably large volume fraction of the unit cell for ion mobility of 72.38% for the given parameters, providing the mechanism for the high ionic intracrystalline proton conduction announced by several authors^3,4,13,32^. The average site-energy computed by bond-valence theory for both mobile protons is − 2.95 eV, differing between both species in only 0.12 eV.

The H2 atom is bonded to the framework in a Sb^5+^‒OH fashion, with hydroxyl interatomic distances H2‒O1 of 1.13(6) Å. The O2 at 32*e* Wyckoff sites is coordinated by three H1 species at 96*g* positions in a tetrahedral arrangement, with internal H1‒O2‒H1 angles of 104.0(16)°. The lone electron pair of the oxygen atoms belonging to the hydronium groups are oriented in the 8*a* ($${\raise0.5ex\hbox{$\scriptstyle 1$} \kern-0.1em/\kern-0.15em \lower0.25ex\hbox{$\scriptstyle 8$}}$$,$${\raise0.5ex\hbox{$\scriptstyle 1$} \kern-0.1em/\kern-0.15em \lower0.25ex\hbox{$\scriptstyle 8$}}$$,$${\raise0.5ex\hbox{$\scriptstyle 1$} \kern-0.1em/\kern-0.15em \lower0.25ex\hbox{$\scriptstyle 8$}}$$) direction. The H1 atoms are located about 1.400(10) Å away from the O1 oxygen of the covalent framework, with which a hydrogen bond is likely established. Remarkably, the mean H1‒O2 interatomic distance within the hydronium unit is 1.323(11) Å, which might seem rather large for a well-established (coordinated) covalent bond. The reason for this is that, statistically, there are 1.20(7) hydronium groups within the framework per formula unit, but in reality, only one or two subunits may fit in each cavity, off-centre of the 8*a* ($${\raise0.5ex\hbox{$\scriptstyle 1$} \kern-0.1em/\kern-0.15em \lower0.25ex\hbox{$\scriptstyle 8$}}$$,$${\raise0.5ex\hbox{$\scriptstyle 1$} \kern-0.1em/\kern-0.15em \lower0.25ex\hbox{$\scriptstyle 8$}}$$,$${\raise0.5ex\hbox{$\scriptstyle 1$} \kern-0.1em/\kern-0.15em \lower0.25ex\hbox{$\scriptstyle 8$}}$$) Wyckoff site and displaced along the (*x*,*x*,*x*) direction. Therefore, about eight cavities hold one single hydronium ion, whereas two H_3_O^+^ subunits must be contained in two of them. In the latter case, owing to the repulsive electrostatic forces between the two highly electronegative O2 atoms fitting close to each other at a mean 1.752(10) Å distance, the H_3_O^+^ units accommodate even farther from the 8*a* site. Due to this shift along the main diagonal, H1 atoms become closer to the O1 atoms of the covalent framework, fostering a resonance in which the H1 protons may jump into the H2 48*f* position and establish a hydrogen bond with their correspondent O2 at a mean distance of 1.679(17) Å. Recently, we have found a similar shift along the main diagonal direction due to the increasing occupancy of H_3_O^+^ groups on a related family of Sb and Te pyrochlore-like acid oxides^31^. Certainly, it was confirmed that the shift of the O′ atoms along this path, regarding the lower-multiplicity 8*a* ($${\raise0.5ex\hbox{$\scriptstyle 1$} \kern-0.1em/\kern-0.15em \lower0.25ex\hbox{$\scriptstyle 8$}}$$,$${\raise0.5ex\hbox{$\scriptstyle 1$} \kern-0.1em/\kern-0.15em \lower0.25ex\hbox{$\scriptstyle 8$}}$$,$${\raise0.5ex\hbox{$\scriptstyle 1$} \kern-0.1em/\kern-0.15em \lower0.25ex\hbox{$\scriptstyle 8$}}$$) Wyckoff site, is frequent within the pyrochlore family^33^.

Therefore, when two hydronium groups get close to each other, the central protons become prone to delocalization. Here, it is very likely that one of these in-between protons starts resonating and becomes a shared ion between the two O2 and the O1 oxygens. Such a scheme would entail the bonding of four protons to two O2, the share of a fifth H^+^ between these and one O1 oxygen, and the sixth remaining H^+^ species leaving the H_3_O^+^ groups behind by following the ionic conduction path and bonding to the opposite O1 at a mean O2***–***H2 distance of 2.811(12) Å. As there are statistically less than 0.8 H2 atoms per cavity, there is room for the proposed mechanism as some of the cages will not present an OH hydroxyl group at all. A schematic representation of this effect is shown in Fig. 2d.

The determined Sb–O1–Sb angle and Sb–O1 distances within the Sb_2_O_6_^2−^ covalent network are 137.33(3)° and 1.9663(12) Å, respectively, which are larger than those of 136.39(2)° and 1.9624(5) Å recently reported for an AA derivative, viz. Sb_6_O_13_, obtained by thermal decomposition of the former^19^. The unit-cell parameter is also longer for AA, about 0.53% more than that of Sb_6_O_13_ (10.36052(15) vs 10.30653(11) Å). A wider and bigger cavity for the hydrated sample is consistent with the presence of 1.20(7) voluminous H_3_O^+^ groups per cage, in contrast with the 0.5 Sb^3+^_2_–O′ groups found in the calcined sample. The main interatomic distances and angles obtained are summarized in Table 2.

**Table 2:** Selected interatomic distance and angles refined from combined SXRD and NPD data. Main interatomic distances and angles for (H_3_O)_1.20(7)_H_0.77(9)_Sb_2_O_6_, with cubic space group $Fd\overline{3 }m$ (*#* 227) and *Z* = 8, from dual SXRD and NPD data refinement collected at 298 K (λ_SXRD_ = 0.44271 Å, λ_NPD_ = 1.5947 Å, Origin Choice # 2). Sb_2_O_6_^2−^ covalent framework, H_3_O^+^ subunit, and non-bonding atoms categories are classified. In the latter, only meaningful distances and angles from atomic pairs and triplets of near non-bonding elements are summarized.

Bond (occurrence)	Distance (Å)	Atoms set	Angle (°)
**(Sb**_**2**_**O**_**6**_^**2−**^**) covalent framework**			
Sb–O1 (× 6)	1.9663(12)	Sb–O1–Sb	137.33(3)
O1–H2 (× 1)	1.12(4)	O1–Sb–O1	180.00(6)
		O1–Sb–O1	87.39(4)
		O1–Sb–O1	92.61(8)
		Sb–O1–H2	111.3(14)
**(H**_**3**_**O**^**+**^**) unit**			
O2–H1 (× 3)	1.323(11)	H1–O2–H1	104.0(16)

Non-bonding pairs (occurrence)	Distance (Å)	Atoms set	Angle (°)
**Non-bonding atoms**			
Sb–Sb (× 6)	3.66307(2)	Sb–O1–O2	98.3(3)
Sb–O1 (× 6)	3.7791(6)	Sb–O1–O2	110.1(3)
Sb–O2 (× 12)	3.845(7)	Sb–O1–H1	109.0(7)
Sb–H2 (× 6)	2.59(3)	O1–O1–H2	135(3)
Sb–H1 (× 12)	2.760(10)	O1–O2–H2	108.7(6)
O1–H1 (× 2)	1.400(10)	O1–H2–O2	149(3)
O1–H1 (× 4)	2.471(9)	O1–H2–O2	92(2)
O1–H1 (× 4)	3.111(9)	O1–H2–H1	129(2)
O1–O2 (× 2)	2.697(7)	O1–H2–H1	88(2)
O1–O2 (× 2)	3.034(7)	O1–H1–O2	164.1(9)
O2–O2 (× 3)	1.752(10)	H1–O1–H2	66(2)
O2–O2 (× 1)	2.340(10)	H1–O2–H2	93.5(9)
O2–H2 (× 2)	1.679(17)	H1–O2–H2	115.3(15)
O2–H2 (× 2)	2.788(7)	H2–H1–O2	99.3(9)
O2–H2 (× 2)	2.811(12)	H2–H2–H2	65.0(15)
H2–H2 (× 4)	2.90(4)	H2–H2–H2	57.5(3)
H2–H2 (× 1)	4.10(6)	O1–O1–O1	107.56(6)
H2–H1 (× 4)	2.250(10)	O1–O1–O1	111.181(16)
H2–H1 (× 2)	3.33(2)		
H2–H1 (× 4)	3.18(3)		
H2–H1 (× 2)	4.02(4)		
H1–H1 (× 2)	2.085(19)		
H1–H1 (× 2)	2.92(2)		

The obtained Rietveld profiles are exhibited in Fig. 1b,c and in more detail in Supplementary Fig. S1. The domain size assessment, determined by Scherrer’s^34,35^ equation from SXRD data, yields a crystallite of 42.00(8) nm average apparent size. Instrumental broadening was deconvoluted for Scherrer's apparent domain size determination, see “Methods” for more details.

### Water and proton content assessment

**Figure 3:**
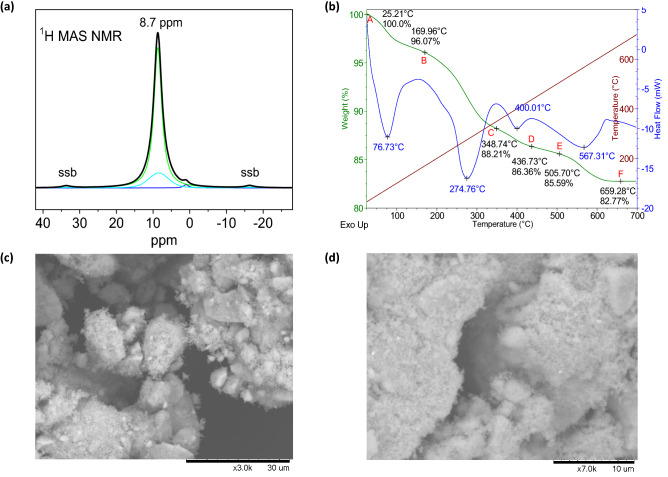
(**a**) ^1^H MAS NMR deconvoluted spectrum, where three contributions can be identified: two corresponding to the H1 and H2 atoms at 8.70 ppm and 8.46 ppm, respectively, and a third, parasitic signal at 1 ppm coming from the rotor cap. The first two present higher chemical shifts than water molecules (5 ppm), accounting for the acidity of the protons of the sample. (**b**) TGA/DSC curves of the thermal analysis of the AA. Labels correspond to the stoichiometric formulae (A) (H_3_O)_1.20_H_0.77_Sb_2_O_6_·0.703 H_2_O or Sb_2_O_5_·2.89 H_2_O, (B) (H_3_O)_1.20_H_0.77_Sb_2_O_6_ or Sb_2_O_5_·2.19 H_2_O, (C) H_1.1_Sb_2_O_5.55_ or Sb_2_O_5_·0.55 H_2_O, (D) H_0.32_Sb_2_O_5.16_ or Sb_2_O_5_·0.16 H_2_O, (E) Sb_2_O_5_, and (F) Sb_6_O_13_. These structures were inferred from the total weight loss of the AA sample, and later confirmed by carefully matching up these curves with the TGA and DTA curves of the Kovalenko et al*.* thermolysis analysis reported for a hydrated antimony pentoxide sample^38^. (**c**,**d**) low magnification SEM images of the AA sample obtained by soft-chemistry oxidative hydrolysis reaction.

The occupancies of O2, H1, and H2 species used on the combined Rietveld refinement were determined by means of MAS NMR applied to dry AA. This is a powerful technique to identify the different ^1^H species that may be found within the crystal structure. Prior to this analysis, the sample was dried at 105–107 °C to eliminate all contributions to the final spectrum that could come from adsorbed water. The resulting deconvolution, shown in Fig. 3a, is mainly made up of two components with different widths and intensities, corresponding to the two proton types observed by Rietveld refinement from NPD data. Additionally, a small component has been introduced to the deconvolution at a chemical shift of 1 ppm, corresponding to a parasitic signal from the organic rotor cap. The chemical shift values of the main two contributions are rather similar to each other (8.70 for H1 and 8.46 ppm for H2) and, in both cases, higher than those of the water molecule (5 ppm), indicating a somewhat stronger acidity of the protons. The dependence of the chemical shift or anisotropy with the orientation regarding the external magnetic field B_0_ is not pronounced, and the calculated isotropic chemical shift (tabulated in Table 3) coincides with the position of the main components. At the same time, proton contributions (H1 and H2) present similar small chemical shift anisotropy (usually termed CSA) patterns, which are slightly broadened by anisotropic interactions, meaning that the structural environment is slightly distorted in both cases. Signal contribution widths, on the other hand, are noticeably different; in NMR, a higher width may indicate less mobility due to greater interaction with the surface and a higher heterogeneity of structural positions. From the two main deconvoluted signals, the wider and less significant one (6.5 ppm wide, 17.49%) is assigned to the more restrained, short-bonded H2 protons, while the narrow and stronger signal (2.5 ppm, 81.30%) corresponds to the H1 protons constituting the high-mobility hydronium groups. The relationship between the two areas is 4.648, in close agreement with the H1/H2 Rietveld SOFs ratio of 4.674. Principal elements deduced from sideband patterns are given in Table 3, where isotropic chemical shift, anisotropy, and asymmetry parameters corresponding to each site are also included. A brief explanation of the tabulated elements is presented in “Methods”.

**Table 3:** ^1^H MAS NMR chemical shift anisotropy data from spectra recorded at 10 kHz. In spectra analysis, position, linewidth, and integrated area of central components were deduced with non-linear iterative techniques. The analysis of spinning sideband patterns enables a determination of the isotropic chemical shift (δiso), and the axiality (dCS) and asymmetry parameter (η) of the chemical shift anisotropy tensor.

Signal	Signal source	Line model	Amplitude	Position (ppm)	Width (ppm)	Integral (%)	$xG/\left(1-x\right)L$	dCS (ppm)	η
#1	H1	CSA MAS	12.62	8.70	2.65	81.30	0.00	10.00	0.50
#2	H2	CSA MAS	1.35	8.46	2.8 (1)	17.49	0.70	15.00	0.50
#3	Rotor cap	Gaus/Lor	0.30	1.00	6.0 (1)	1.21	0.00	–	–

In the AA series, moisture is frequently present, and the total water amount they contain seems to vary from zero to six H_2_O molecules per formula unit^1,5,17,28^, some of which are expected to be adsorbed on the solid surface linked by H bonds to the outmost framework oxygen atoms and exposed acid groups. In order to assess this moisture content and to confirm the crystalline water determined by MAS NMR, a TGA/DSC analysis was performed on both, a dry, and a long-term air-stored sample. The weight and heat flow curves are summarized in Fig. 3b.

The dry sample was used to determine the quantity of hydronium and H2 species, obtaining values in close agreement with those derived from NMR and in agreement with the proposed structural model. It comes to no surprise that AA presents some major water affinity, as a small amount of moisture was adsorbed on the dry sample during the TGA/DSC experiment setup, and later degassed in the heating process up to 150 °C. Seamlessly, this extraordinary water affinity was recently identified and reported by our group for a potassium pyrochlore-like niobate and tungstate^33^, probably bound to many series of pyrochlore-like materials as a common behaviour pattern. For the assessment of H content in the dry sample, this last moisture-related contribution was deducted. The final stoichiometric formula achieved with TGA/DSC for dry AA is (H_3_O)_1.24_H_0.76_Sb_2_O_6_, in agreement within the standard deviations with that obtained from the structural refinement. Moreover, the dehydrated sample corresponds to a stoichiometric formula close to Sb_2_O_5_·2.19 H_2_O, which is fairly similar to the one reported by Ozawa et al*.* (Sb_2_O_5_·2 H_2_O)^3^, pioneers in the soft-chemistry procedure utilized here to synthesize the AA phase. By combining the refined crystallographic formula with the TGA results, 0.703 moisture water molecules were determined for the moisturized and long-term stored AA sample, which can be described as Sb_2_O_5_·2.88 H_2_O. Moisture water molecules are presumed to be physisorbed on the surface of the AA crystals through hydrogen bond linkages^1,3,36^. The thermal decomposition steps that AA experiences upon warming up have been widely studied along the years^6,7,37^. Perhaps the most significant contribution in this regard is the one made by Kovalenko et al*.* in 2018 by studying the thermal decomposition of hydrated antimony pentoxide, obtained by oxidation of antimony(III) chloride in nitric acid and later hydrolysis in water. Authors’ assessment was performed by thermal analysis in combination with mass spectrometry analysis of released gases, and reliable intermediate steps were presented. Our TGA/DSC study is in close agreement with the reported decomposition steps, and similar intermediate formulas were labelled for each meaningful step in its corresponding figure.

### Scanning electron microscope

SEM images shed light on the microscopic uniformity of the of AA. This landscape is compatible with the soft-chemistry procedure here used to synthesize the solid. Figure 3c,d shows two pictures of the long-term stored sample, displaying particles of a size smaller than a micron. This is in line with the colloidal nature of the sample, and the impossibility of collecting it from the slurry by a simple filtration process.

### Structural short-range order studies

**Figure 4:**
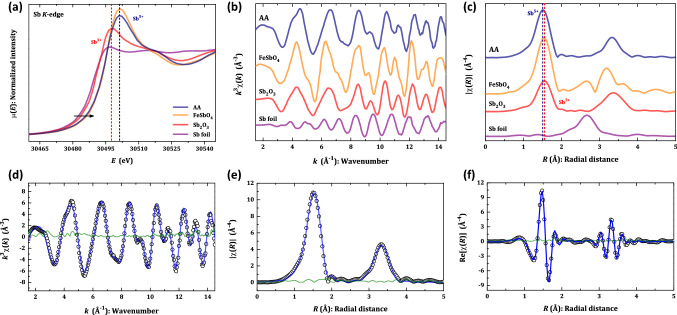
Structural short-range order studies. Room condition Sb *K*-edge XANES spectra of AA as compared with reference samples of Sb foil, Sb_2_O_3_, and FeSbO_4_ (**a**). The *k*^3^-weighted EXAFS signals (**b**) and their corresponding moduli of the Fourier transform (**c**). The fitting was performed using the scattering paths in Table 4: the EXAFS oscillations *k*^3^χ(*k*) (**d**), moduli of the Fourier transform |χ(*R*)| (**e**), and its real part Re[χ(*R*)] (**f**) in *R* space. The open symbol stands for the experimental point, the solid blue line is the best fit, and the green line the residue.

XAS is a powerful tool to probe both chemical features of the constituting elements, such as valence and coordination environment around the target atoms, and local structural information, which includes average nearest-neighbour distances and coordination number^39^. XANES (X-ray absorption near-edge structure) part of the XAS spectra provides information on the valence state as the edge position, such that the binding energy of the bound electrons increases with the valence^40^. Figure 4a compares the XANES at Sb *K*-edge (30.491 keV) of AA with the reference samples, including the Sb foil (Sb^0^), Sb_2_O_3_ (Sb^3+^) and FeSbO_4_ (Sb^5+^). One may see the blue shift of the edge position when the valence state increases from 0 up to 5+, as indicated by the black arrow. The shift Δ*E* of the edge energy, from the Sb^0^, is 1.7 eV for valence Sb^3+^, while for valence Sb^5+^ of AA is 5.6 eV. Such a result agrees with the edge shift observed for FeSbO_4_ oxide, which also contains Sb^5+^. It is also worth noting that the XANES features of AA increase as compared to that one of Sb_2_O_3_, meaning that the coordination number of the first shell Sb–O has also risen.

Quantitative information on the local structure was obtained using the extended part of the XAS spectra, the so-called EXAFS (extended X-ray absorption fine structure). Indeed, EXAFS spectra were recorded up to *k* = 16 Å^−1^, as represented in Fig. 4b. Here, the EXAFS oscillations *k*^3^χ(*k*) of Sb foil, Sb_2_O_3_, FeSbO_4_, and AA are compared, showing similarities among the oxides. This can be better seen by plotting the moduli of the Fourier transform in *R* space in Fig. 4c: two main peaks at 1.52 and 3.35 Å (not corrected by photoelectron phase-shift). In order to evaluate the pair-bond distances and the coordination numbers, the standard EXAFS equation was used, which stands for^39^:

1 $$\chi (k,\Gamma )=\frac{{N}_{\Gamma }{S}_{0}^{2}}{2k{R}_{\Gamma }^{2}}\cdot {F}_{\Gamma }(k,{R}_{\Gamma })\cdot sin\left[2k{R}_{\Gamma }+{\varphi }_{\Gamma }(k)\right]\cdot {e}^{-2{\sigma }_{\Gamma }^{2}{k}^{2}}\cdot {e}^{-\frac{2{R}_{\Gamma }}{\lambda (k)}}$$

such that ${R}_{\Gamma }$, ${N}_{\Gamma }$, and ${\sigma }_{\Gamma }$ are the structural parameters to be determined for each photoelectron path: distance from absorber atom to its neighboring ones, coordination number of the shell, and Debye–Waller (DW) factor (it measures the mean square relative displacement), respectively. ${S}_{0}$ stands for the amplitude reduction factor from a previous calibration using a metallic antimony (${S}_{0}$ is equal to 0.7802 for all the samples). ${F}_{\Gamma }(k,{R}_{\Gamma })$, $\lambda (k)$, and ${\varphi }_{\Gamma }(k)$ denote the backscattered amplitude, photoelectron mean free path, and phase shift, correspondingly, which are determined by the FEFF-8 code^41^.

The EXAFS signal at room temperature of AA was fitted using three neighbouring shells: one short Sb–O, one Sb–Sb, and a long Sb–O one. All the adjusted parameters of interest in Eq. (1) are listed in Table 4. Figure 4d–f shows the quality of fitting by exhibiting the EXAFS oscillations, moduli of the Fourier transform and its real part in *R* space, respectively. The first peak in |χ(*R*)| at 1.52 Å (not phase-shift corrected) denotes the Sb^5+^–O bond with *R*_Sb−O_ = 1.955(1) Å and coordination number *N*_Sb−O_ around 6.5(1). Such a shell fully agrees with the pair bond Sb−O1 (× 6) with a distance of 1.9663(12) Å, as obtained from NPD. The second feature in |χ(*R*)| at 3.35 Å has two components: a metallic-metallic pair with *R*_Sb−Sb_ = 3.612(1) Å and a metallic-anion one with *R*_Sb−O_ = 3.898(1) Å. The Sb–Sb pair possesses a coordination environment close to 3, which may be associated with the non-bonding pair Sb–Sb (× 6) with a metal–metal distance of 3.66307(2) Å in Table 2. The second non-bonding pair Sb–O has *R*_Sb−O_ = 3.898(1) Å and coordination number *N*_Sb−O_ around 6.0(1), being similar to that obtained for Sb–O2 (× 12) with a distance of 3.845(7) Å. Details on the EXAFS analyses in Sb_2_O_3_ and FeSbO_4_ samples can be found elsewhere^19^.

**Table 4:** Structural parameters extracted from EXAFS data. ${R}_{\Gamma }$ is the distance from absorber atom, ${N}_{\Gamma }$ is the average coordination number, ${\sigma }_{\Gamma }^{2}$ the Debye–Waller factor, and *R*-factor stands for the quality factor of the fitting. Maximum number of independent variables as imposed by the uncertainty principle: *N*_idp_ ≈ 2Δ*k*ΔR/π. *N*_var_, number of variables used during the fitting procedure.

Sample	Shell	${R}_{\Gamma }$, Å	${N}_{\Gamma }$	${10}^{-3}\times {\sigma }_{\Gamma }^{2}$, Å^2^
Antimonic acid	Sb–O	1.955(1)	6.5(1)	3.4(6)
Edge position, eV	Sb–Sb	3.612(1)	2.8(1)	2.7(8)
30,495.6	Sb–O	3.898(1)	6.0(1)	12(1)

*N*_idp_	*N*_var_	R-factor	Δ*k*-range, Å^−1^	Δ*R*-range, Å
26	8	0.0115	2–14.5	0.9–4.2

Differently from NPD, here it is not possible to identify pair containing this hydrogen by EXAFS, since the atomic number of Hydrogen is 1; therefore, the shortest and longest distances are missing in this study. The DW factors of the shells Sb–O (1.955(1) Å) and Sb–Sb (3.612(1) Å) were derived as 3.4(6) and 2.7(8) × 10^−3^ Å^2^, respectively. These values agree well with those reported for cubic Sb_2_O_5_, meaning that AA and the last oxide have a very similar short-range structure^42^, i.e. with a similar covalent framework composed by Sb^5+^–O pair bonds.

## Conclusions

A compelling study of the so-called ‘antimonic acid’ structure by robust local- and long-range techniques, together with a BVEL analysis, shed light on the stoichiometry and atomic distribution in the crystal structure, and provide a plausible ionic diffusion mechanism for its well-established high proton conductivity. The structure can be defined as a defect pyrochlore, belonging to the $Fd\overline{3 }m$ space group. Aided by a combined Rietveld refinement from SXRD and NPD data, and by TGA/DSC and MAS NMR studies, we were capable of identifying two main types of protons in this material, one at 96*g* Wyckoff sites that belongs to the highly delocalized hydronium subunits and presents lengthened prolate displacement ellipsoids, and another one at 48*f* positions, directly bonded at 1.12(4) Å to the oxygen atoms constituting the *B*_2_O_6_^−2^ covalent framework within which the hydronium groups percolate. The refined crystallographic formula is (H_3_O)_1.20(7)_H_0.77(9)_Sb_2_O_6_, with 0.703 water molecules from physisorbed moisture per formula unit. We found neither additional H species nor the presence of Sb^3+^ remaining from the oxide precursor or generated by reduction of Sb^5+^ at *A* sites. Although no additional crystallization water was found, a plausible mechanism where two H_3_O^+^ groups within the same cavity are prone to become two H_2_O molecules and two H2 protons is proposed, endorsed by the repulsion among hydronium units sharing the same cavity, and by the 48*f* site availability. Good Rietveld refinement reliability factors were achieved, and the XAS, the MAS NMR, and TGA/DSC results are all consistent with the proposed model. In particular, XANES endorsed the pentavalent state of antimony ion in AA, while EXAFS probed the covalent framework composed by Sb^5+^ and O1 atoms.

## Methods

### Sample preparation

All the commercially available *ReagentPlus* or Analytical-grade reagents were purchased at Sigma Aldrich and Fisher Scientific.

Antimonic acid was obtained by an oxidative hydrolysis soft-chemistry reaction. It begins from Sb_2_O_3_ and a 31% H_2_O_2_ solution, following previously described procedures^3,43^. The mixture was stirred at 343 K for 24 h, while the hereunder reaction occurs:

2 $$S{b}_{2}{O}_{3}+2\hspace{0.17em}{H}_{2}{O}_{2}+{\left(p-1\right) H}_{2}O\stackrel{ }{\to }{\left({H}_{3}O\right)}_{p}{H}_{2-p}S{b}_{2}{O}_{6}.$$

The white colloidal slurry is centrifuged at 15,000 rpm for 10 min until nearly complete sedimentation. The product, a glassy-white solid, is then dried in air at 105–107 °C for 48 h and finally ground.

The (H_3_O)_*p*_H_2−*p*_Sb_2_O_6_ sample was firstly investigated utilizing X-ray powder diffraction (XRD). Laboratory XRD data were collected with a conventional diffractometer (40 kV, 30 mA) in Bragg–Brentano reflection geometry with Cu K_α_ radiation (λ_mean_ = 1.5418 Å). The SXRD pattern was collected at the *CELLS‒ALBA* facility, Barcelona (Spain), in the MSPD high-angular resolution diffractometer under an incident beam with an energy of 28 keV and a wavelength of λ = 0.44271 Å. For determining the instrumental broadening, the sample was characterized together with a powdered Na_2_Ca_3_Al_2_F_14_ fluoride (NAC) standard. The high-angular resolution mode (MAD set-up) was used on the MSPD-diffractometer^44^. The polycrystalline powder was contained in a spinning glass capillary of 0.7 mm diameter. For the NPD experiments, the D2B high-resolution two-axis diffractometer was used, installed at the Institut Laue-Langevin, in Grenoble (France). The sample (about 2–3 g) was contained in a vanadium can. The full diffraction patterns were collected in a 2 h-long analysis time. A wavelength of 1.5947 Å was selected from a Ge monochromator; the measurement temperature was 298 K.

### Structural refinement from synchrotron X-ray and neutron diffraction data

*FULLPROF*^45^ software (see “Methods”) was used for performing the combined Rietveld refinement^46^ from SXRD and NPD data. A relative pattern weight of 0.10/0.90 favouring NPD data was considered, as it presents an absence of form factor and exceptional sensitivity for both hydrogen and oxygen atoms, essential for determining their positions, occupancies and atomic displacement factors (ADPs). Moreover, H^+^ atoms are invisible to X-rays and the atomic weight ratio between Sb and O is large enough for the SXRD to differentiate them even at low pattern weighting. The best ADPs and lowest Rietveld reliability factors were obtained for the announced relationship.

The Thompson–Cox–Hastings^47^ pseudo-Voigt convoluted with axial divergence asymmetry over SXRD data were used to determine the crystallite size. A calculated *μR* = 0.92 absorption correction coefficient determined by adopting a 0.5 packed factor was included in the refinement for compensating transmission and absorption of the X-rays through the irradiated cylindrical volume of the sample. An apparent isotropic crystallite size of 42.00(8) nm and average maximum generalized strain of *ε* = 6.666(7) × 10^–4^ were obtained through microstrain- and domain size-determining *FULLPROF* modules. For the NPD data treatment, a pseudo-Voigt^48^ function with the asymmetry correction published by Berar and Baldinozzi^49^ were respectively employed for the simulation of the peak shape and the asymmetry assessment.

Both SXRD and NPD backgrounds were linearly interpolated between 68 and 44 individual refined points. The coherent neutron scattering lengths used in the Rietveld refinement are internally tabulated in the program *FULLPROF*, rated in 5.570, 5.803, and − 3.739 fm for Sb, O, and H atoms, respectively.

BVEL was carried out with *BondStr*^50^ software (see “Methods”), a module embedded in the *FULLPROF* toolbar. For ensuring a percolation energy convergence of a few hundredths of electron volt, BVEL analysis considering H^+^ as the mobile ion was computed by applying a grid resolution of 0.1 Å and a percolation radius of 8 Å, suiting with the optimal parameters described by Katcho et al*.* in their high-throughput BVEL calculation of Li and Na ionic conductors^50^. As both mobile ion share almost the same minimum site-energy (− 2.93 and − 3.05 eV for H1 and H2, respectively), and that they fit in the same conduction path, we tacitly assume that the migration energy *E*_*m*_ is equivalent to the threshold energy *E*_*th*_ here determined, defined as the energy at which the proton pathway starts percolating across the unit cell^50^. Hence, undertaking a multiple-technique approach, we disclose a detailed and comprehensible structural description of the AA, which is compatible with its main chemical properties.

### Magic-angle spinning nuclear magnetic resonance spectroscopy

The chemical shift anisotropy is the interaction between the external magnetic field B_0_ and the electron density surrounding the nucleus, owing to the magnetic moment coming from its rather ellipsoidal shape. The weak secondary magnetic fields that are generated are added or subtracted to B_0_, modifying the magnetic field around the nucleus, and therefore its resonance frequency in a so-called ‘shielding’ process that results in a chemical shift. The three main values of the shielding associated tensor are frequently expressed as a function of the isotropic chemical shift (δiso), and the axiality (dCS) and asymmetry parameter (η) of the chemical shift anisotropy tensor. For nucleus in an axial symmetry site, it is true that δ*xx* = δ*yy* ≠ δ*zz* and η = 0. The shape of the powder sample line is very different, depending on the symmetry of both the shielding tensor and the site where the nucleus is located. The MAS experiment (‘magic angle spinning’ or rotation of the sample around the magic angle) can average the chemical shift anisotropy. Even slow spinning provides very narrow lines, although there may be a substantial number of spinning sidebands. It is interesting to note that the orientation-dependent information of the interaction remains, embedded in the amplitudes of the spinning sidebands. Indeed, a simulation can extract the principal values of the chemical shift anisotropy from slow speed MAS spectra.

^1^H MAS NMR spectrum was recorded on a Bruker AVANCE 400 spectrometer. Single pulse sequences were used to irradiate the sample at the ^1^H resonance frequency in a 9.4 T (400 MHz) magnetic field. The sample was placed on zirconia rotors that rotate inside the probe at an angular frequency of 10 kHz around the magic angle (54° 44′ with respect to the external magnetic field). NMR spectrum was obtained after excitation of the sample with a π/2 pulse duration of 4.7 µs and an interval between successive accumulations of 5 s. The total number of accumulations was 72. To determine the values of the chemical shift, tetramethylsilane was used as internal standard. By adding the components as a combined Lorentzian–Gaussian form, a calculated envelope that reproduces the spectrum is obtained, from which the intensity, position (chemical shift) and width parameters of central components were deduced with non-linear iterative techniques (DMFIT software^51^, see “Methods”). The analysis of spinning sideband patterns enables a determination of chemical shift, dCS and η parameters.

### X-ray absorption spectroscopy at the *CLÆSS *beamline of the *ALBA* synchrotron

The X-ray absorption process was performed by measuring the photon flux through three ionization chambers. This well-established technique used in transmission mode provided an exact measurement of the X-ray absorption coefficient. The resulting absorption spectra were then characterized by one or more jumps (absorption edges), whose energy positions are element specific since they coincide with the energy of the corresponding atomic core level. The X-ray transitions are controlled by the dipolar selection rules relating to well-defined atomic symmetry of the involved core hole and the final state angular momenta. XANES spectra show a remarkable site-specific behaviour, because they are sensibly affected by the strong spatial localization of the initial core–shell state.

Short-range atomic studies were performed by means of XAS at the BL22-CLÆSS beamline of the Spanish synchrotron, CELLS-ALBA, Barcelona, with electron energy and current in the ring of 3 GeV and 200 mA, respectively. Data acquisition was performed with a double crystal monochromator with two Si(311) crystal pairs and three ionization chambers for determining the photon flux before/after the sample and before/after the metal foil employed for energy calibration. In this way, the X-ray absorption coefficient may be exactly measured. Details on the beamline setup can be found elsewhere^52^. Concerning the sample preparation for XAS measurements, the samples were ground in an agate mortar with an inert matrix (boron-nitride, BN), pelletized into disks to optimize the absorption jump of the XANES spectrum, and then protected with Kapton tape. The reference samples such as Sb foil (> 95%) and Sb_2_O_3_ (99.7%) were purchased from Aldrich and Alfa Aesar, respectively. FeSbO_4_ was synthesized using solid-state reaction method, as detailed elsewhere^19^.

### Complementary techniques

The TGA/DSC characterization was performed in a Mettler TA3000 system equipped with a DSC Q-100 unit. The measurements were performed in heating runs from room temperature to 700 °C with a rate of 10 K min^−1^ for powder samples encapsulated in standard alumina crucibles. About 49 and 56 mg of sample were used for the dry-basis and long-term stored AA experiments, correspondingly. The thermal decomposition reaction of the AA is determined as follows:

3 $$3 \left( {H_{3} O} \right)_{p} H_{2 - p} Sb_{2} O_{6} \cdot n H_{2} O\mathop{\longrightarrow}\limits^{\Delta }Sb_{6} O_{{{13}}} + O_{2} + \left( {3 + 3p + n} \right)H_{2} O.$$

SEM experiments were conducted with a Hitachi TM1000 (Hitachi High‑Technologies Corporation, Minato, Tokyo, Japan) desktop instrument with an acceleration voltage of 1.5 kV and a 90 s acquisition time.

### Details on data processing

*FULLPROF*^45^ toolbar software (Version 5th May 2020, Institut Laue-Langevin Grenoble, France, https://www.ill.eu/sites/fullprof/) includes the embedded modules *WinPLOTR-2006* (Version 0.50 of June 2013, Institut Laue-Langevin Grenoble, France), *BondSTR* (Version July 2010, Institut Laue-Langevin Grenoble, France) and *GFOURIER* (Graphic Fourier Program, Version 04.06 of 2007, Univ. La Laguna, Tenerife, Spain), that were used for the Rietveld Refinement, the Fourier difference density maps calculation, and the BVEL analysis, respectively. Data processing was performed with *OriginPro* (Version 8 SR0 and 2018 SR1, OriginLab, Northampton, MA, USA, https://www.originlab.com). Crystal structure projections were generated using *VESTA*^53^ (Visualization for Electronic and STructural Analysis, Version 3.5.5 of 26th September 2020, 64-bit Edition, https://jp-minerals.org/vesta/en/) graphing tools. ATHENA and ARTEMIS from the *Demeter* suite^41^ (Version 0.9.26, 64-bit Edition, https://bruceravel.github.io/demeter/) were employed to process the XAFS data. MAS NMR spectra were treated with the *DMFIT* software^51^ (Version 2020.03.06, https://nmr.cemhti.cnrs-orleans.fr/dmfit/).

## Supplementary Information

Supplementary Information 1.

Supplementary Information 2.

## Data Availability

The datasets generated during and analysed during the current study are available from the corresponding author on reasonable request.
