# Supplementary material for: Synergy of diffraction and spectroscopic techniques to unveil the crystal structure of antimonic acid
Source: Sci Rep. 2021 Sep 7;11:17763. doi: 10.1038/s41598-021-97147-0 (PMC8423724; doi:10.1038/s41598-021-97147-0)
Supplement: Supplementary file 1 — Supplementary Information 1. [file 41598_2021_97147_MOESM1_ESM.docx]

**SUPPLEMENTARY INFORMATION**

**Synergy of diffraction and spectroscopic techniques to unveil the crystal structure of antimonic acid**

S.F. Mayer,^1*^ J.E. Rodrigues,^1,2^ I. Sobrados,^1^ J. Gainza,^1^ M.T. Fernández-Díaz,^3^ C. Marini,^4^ M.C. Asensio,^1^ J.A. Alonso^1^

*^1^ Instituto de Ciencia de Materiales de Madrid (ICMM), Consejo Superior de Investigaciones Científicas (CSIC), Sor Juana Inés de la Cruz 3, E‑28049 Madrid, Spain.*

*^2^ European Synchrotron Radiation Facility, ESRF, 71 Avenue des Martyrs, 38000 Grenoble, France.*

*^3^ Institut Laue Langevin (ILL), BP 156X, F-38042 Grenoble, France.*

*^4^ CELLS−ALBA Synchrotron, Cerdanyola del Valles, E-08290 Barcelona, Spain.*

∗To whom correspondence should be addressed: [*s.mayer@csic.es*](mailto:s.mayer@csic.es)

*Rietveld refinement procedure*

The initial refinement involved every atom other than H in the structure, namely Sb, O1, and O2 at 16*d* (½,½,½), 48*f*(*x*,⅛,⅛), and 32*e* (*x*,*x*,*x*) Wyckoff sites, respectively. The arrangement of the first two species constitutes the basic (*B*_2_O_6_)^2−^ covalent pyrochlore framework, while the O2 atoms correspond to the O’ in the theoretical A_2_O’ subunit. Individual isotropic displacement “thermal” factors were initially considered for each atom. The unit cell was then updated by incorporating a single hydrogen atom H1 at 96*g* (*x*,*x*,*z*) special positions [Case A], in concomitance with our recent experience with a related family of hydrated defect pyrochlore-type acidic oxides containing antimony and tellurium^1^. Those solids, of general formula (H_3_O)_1+_*_p_*Sb_1+_*_p_*Te_1−_*_p_*O_6_, presented a single kind of H atom bonded to the O2 atoms at distances close to 1.15 Å, constituting the hydronium acid subunits. After refining the H1 site occupancy factor (SOF), the phase still exhibited an absence of 1.26 positive charges per formula unit. Although good reliability factors were obtained, the corresponding formula was close to (H_3_O)_1.05_Sb_2_O_6_O’_0.15_, lacking electroneutrality. Further tests included constraining the SOFs so that the model achieves an electrically neutral structure [Case B], shown in Supplementary Fig. S2a, but the resultant hydronium subunit reached unrealistically high Debye‑Waller (DW) equivalent isotropic displacement (*U_eq_*) factors, namely those of 0.28(3)×10^-2^ Å^2^ for the H1 and 0.193(9)×10^-2^ Å^2^ for O2 species. As reference, this last value was above 15 times larger than the *U*_eq_ of O1 atom.

As hydrogen nuclei possess a negative nuclear scattering length, a Fourier difference density map would shed light on the regions within the unit cell experiencing a lack of protons as a negative peak of nuclear density. To this purpose, the RT NPD collected data contributed with great value owing to its great sensitivity to H atoms. Among other low-significance difference densities found centred at high-symmetry sites, two spots where H atoms could be present were identified: the 8*a* (⅛,⅛,⅛) special Wyckoff site, and the 48*f* (*x*,⅛,⅛) position, both marked with light blue negative density isosurfaces in Supplementary Fig. S2b. The first site was considered and later discarded after confirming that the SOF of an H atom there included lowered and converged to close-to-zero values (within the standard deviation) during refinement. This negative density cloud is likely a consequence of the high symmetry of the site, since highly regular crystalline cells, such as pyrochlore-type cubic cells, present relatively low reflection counting and a certainly great degree of peaks overlapping. When introduced in the 48*f* site, however, a H atom directly linked to the framework in a Sb^5+^‒OH fashion proved to fit correctly and to enhance the charge neutrality of the crystal. Moreover, the hydroxyl interatomic distance of 1.13(6) Å established between H2 and O1 is in close agreement with crystallized inorganic acids belonging to the same periodic group, such as phosphoric^2^ (0.934 Å) and arsenic^3^ (1.005 Å) oxoacids, and with other high-acidity molecules such as nitric (0.9412 Å) and sulfuric (0.97 Å) acids^4^. The SOFs of H1, H2, and O2 atoms were constrained to each other, according to the results of the NMR analysis of the sample. This last scheme was then used for a final Rietveld refinement by combining the NPD together with SXRD data, also collected at RT. Our findings, displayed in Supplementary Fig. 2c presents some level of similarity with a report published by Slade et al*.* in 1996 on the same crystalline phase^5^. The authors found a hydrogen atom located at 48*f* Wyckoff site, 1.22 Å away from the covalent network oxygen (O1). However, they considered an additional O-H atomic pair located off-centre of the 8*a* site and aligned along the 32*e* (*x*,*x*,*x*) main diagonal, arranged in a tetrahedral unit that is intercalated to the one constituted by the O2 and H2 species (H_3_O^+^ unit) here defined (close‑up as orange-blue atomic pair in Supplementary Fig. S2d). Our attempts to include and refine additional hydronium molecules converged in low-quality agreement factors. Furthermore, water molecules were not found as such within the refined structure, in contrast with Slade et al*.* results^5^. Both disagreements may rely on the different synthesis methods used in each case, and the collected data quality. The low peak signal-to-noise ratio NPD data collected by Slade et al*.* corresponds to a solid that was obtained by digestion of potassium antimoniate in HNO_3_ rather than by following the soft-chemistry oxidative hydrolysis procedure utilized here, previously described by Ozawa et al^6^.

Once the model we used was consistently defined, advanced refinements incorporated anisotropic displacement factors of each atom with the only exception of the H2 species [Case C], as this led to unrealistic negative anisotropic factors.

**Supplementary Figure S1.** Diffraction patterns and structural representation of AA. (**a** and **b**) Plots of combined Rietveld refinement from SXRD and NPD data of (H_3_O)_1.20(7)_H_0.77(9)_Sb_2_O_6_. Experimental (red crosses), theoretical (solid black line), and difference (solid blue line at the bottom) (**a**) SXRD (λ_SXRD_ = 0.44271 Å) and (**b**) NPD (λ_NPD_ = 1.5947 Å) patterns, with Bragg reflection positions marked by vertical green bars.

**Supplementary Figure S2.** Views throughout the Rietveld structure refinement progress. (**a**) Case B scenario, where the H_3_O^+^ subunits occupancy is constrained so that the final crystal presents electroneutrality. This model results in unrealistically high DW isotropic displacement factors. (**b**) Fourier density difference negative isosurfaces from NPD data of the (H_3_O)_1.20(7)_H_0.77(9)_Sb_2_O_6_ sample, collected at RT for the Case B refined model. Two negative spots are highlighted, one at 8*a* Wyckoff sites, which would be generated due to their high site symmetry, and one at 48*f* positions, where a H2 atom could fit. (**c**) Case C and final iteration of the Rietveld refinement, prior anisotropic definition of the atomic displacement factors. (**d**) Close‑up of the model that Slade et al*.*^5^ proposed in 1996 for the antimonic acid phase from the structure Rietveld refinement from NPD data collected at RT. The NPD diffractogram used had a rather low peak signal-to-noise ratio. Although similar to the model we chose in Case C, the authors included an additional H−O atomic pair that we conclude are not present in this material, or their abundance is negligible.

**Caption for the Animated Figure (Supplementary File 2).** Animation of H_3_O^+^ units and H2 species within the cavity created by the framework constituted by SbO_6_ corner‑sharing octahedra. Here, a clearer sight of the structure is displayed. Sb and O atoms of the covalent framework shape the pink octahedra, while the acid groups fit within its cavities with high atomic mobility. Statistically, 1.20(7) hydronium units and 0.77(9) H2 atoms are actually present in each cage. O2 atoms (green) are displaced off‑center trough the main diagonal (*x*,*x*,*x*), bonded to three H1 atoms at 96*g* Wyckoff sites with a mean distance of 1.323(11) Å and in an almost tetrahedral arrangement, with 104.0(16)° angles. H2 atoms (golden) are bonded to the O1 species (red) at a distance of 1.12(4) Å, 1.400(10) Å away (on average) from the oxygen of the H_3_O^+^ subunit.

**REFERENCES**

1. Mayer, S., Falcón, H., Fernandez-Diaz, M. T., Campos-Martin, J. & Alonso, J. A. Structure-properties relationship in the hydronium-containing pyrochlores (H3O)1+pSb1+pTe1-pO6 with catalytic activity in the fructose dehydration reaction. *Dalt. Trans.* **49**, 11657–11667 (2020).

2. Souhassou, M., Espinosa, E., Lecomte, C. & Blessing, R. H. Experimental electron density in crystalline H3PO4. *Acta Crystallogr. Sect. B* **51**, 661–668 (1995).

3. Orosel, D., Dinnebier, R. E., Balog, P. & Jansen, M. The crystal structure of a new mixed valence arsenic(III,V)oxoacid H 6As3+7As5+7O31. *Zeitschrift fur Krist.* **222**, 321–325 (2007).

4. Graner, G. *et al.* 2 Inorganic Molecules. Part 4. in *Inorganic Molecules* 283–359 (Springer-Verlag, 2005). doi:10.1007/10529543_6.

5. Slade, R. C. T., Hall, G. P., Ramanan, A. & Prince, E. Structure and proton conduction in pyrochlore-type antimonic acid: A neutron diffraction study. *Solid State Ionics* **92**, 171–181 (1996).

6. Ozawa, Y., Miura, N., Yamazoe, N. & Seiyama, T. Proton Conduction in Thermally Treated Antimonic Acid Samples. *Chem. Lett.* 1741–1742 (1982) doi:10.1246/cl.1982.1741.
