# Supplementary figures and images for: Synergy of diffraction and spectroscopic techniques to unveil the crystal structure of antimonic acid
Source: Sci Rep. 2021 Sep 7;11:17763. doi: 10.1038/s41598-021-97147-0 (PMC8423724; doi:10.1038/s41598-021-97147-0)

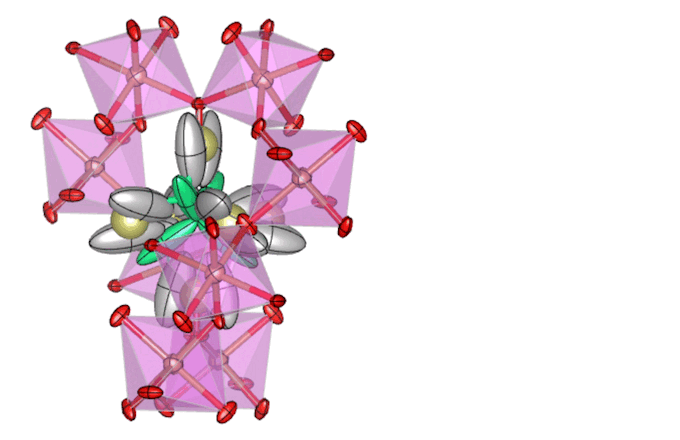

Supplement: Supplementary file 2 — Supplementary Information 2. [file 41598_2021_97147_MOESM2_ESM.gif]
